# Glymphatic dysfunction associates with regional white matter hyperintensities and plasma amyloid-β burden across the Alzheimer’s disease continuum

**DOI:** 10.1017/S0033291726105005

**Published:** 2026-07-07

**Authors:** Hui Juan Chen, Yihao Guo, Weiyuan Huang, Anquan Hu, Tao Liu, Feng Chen

**Affiliations:** 1 Department of Radiology, Hainan General Hospital, Hainan Affiliated Hospital of Hainan Medical University, Haikou, China; 2 Department of Neurology, Hainan General Hospital, Hainan Affiliated Hospital of Hainan Medical University, China

**Keywords:** Alzheimer’s disease, cerebral small vessel disease, diffusion tensor imaging analysis along perivascular spaces, white matter hyperintensities

## Abstract

**Background:**

Glymphatic system dysfunction has been increasingly implicated in Alzheimer’s disease (AD), yet its relationships with cerebral small vessel disease (CSVD), plasma biomarkers, and cognitive impairment across the AD remain incompletely understood.

**Methods:**

We prospectively recruited 216 participants from Hainan General Hospital, including healthy controls (HC), individuals with subjective cognitive decline (SCD), mild cognitive impairment (MCI), and AD dementia. All participants underwent brain magnetic resonance imaging, plasma biomarker testing, and neuropsychological assessments. White matter hyperintensity (WMH) volume from T2-weighted fluid-attenuated inversion recovery images served as a marker of CSVD. The diffusion tensor image analysis along the perivascular space (DTI-ALPS) index assessed glymphatic function. Plasma amyloid β-protein (Aβ) concentrations measured peripheral Aβ levels as a surrogate indicator of amyloid pathology.

**Results:**

The ALPS index was significantly lower in AD patients compared with HC, SCD, and MCI groups (all *P* < 0.01) and tended to be lower in the MCI group relative to SCD. After controlling for demographics and APOE4 status, ALPS positively correlated with the plasma Aβ42/Aβ40 ratio (*r* = 0.16, *P* = 0.038). ALPS index showed significant negative correlations with log-transformed juxtaventricular and juxtacortical WMH volumes (*r* = −0.32, *P* < 0.001; *r* = −0.19, *P* = 0.010), with marginal correlation for periventricular WMH (*r* = −0.13, *P* = 0.052).

**Conclusion:**

Plasma Aβ levels and regional WMH burden are associated with glymphatic dysfunction as indicated by reduced ALPS. Impaired glymphatic clearance also correlates with cognitive impairment, providing theoretical support for novel pathophysiological hypotheses and potential therapeutic targets in AD pathogenesis.

## Introduction

The extracellular accumulation of amyloid-β (Aβ) is a pathological hallmark of Alzheimer’s disease (AD), typically beginning more than 15 years before the onset of dementia symptoms (Sperling, Karlawish, & Johnson, 2013). This abnormal deposition results from impairment of multiple brain clearance mechanisms (Hampel et al., 2021). Rodent studies have shown that the brain glymphatic system is a key pathway involved in Aβ clearance from the brain (Hampel et al., 2021). Animal studies have found that glymphatic dysfunction is associated with Aβ accumulation(Peng et al., 2016; Simon et al., 2022), and human population studies have also demonstrated impaired glymphatic function in AD patients, suggesting that glymphatic function may contribute to AD-related cognitive dysfunction (Hsu et al., 2023). However, research on factors affecting glymphatic function in AD remains limited.

Alzheimer’s disease pathology commonly coexists with cerebral small vessel disease (CSVD), which is a common pathogenic mechanism contributing to AD. Studies show that over 80% of AD patients have coexisting small vessel disease (Toledo et al., 2013). White matter hyperintensities (WMH) are commonly used as imaging markers to represent CSVD. Previous studies have shown that WMH are associated with other glymphatic biomarkers such as enlarged perivascular spaces and choroid plexus volume (Huang et al., 2021; Li et al., 2023). However, most studies have only examined the relationship between total cerebral WMH volume and the glymphatic system, and it remains unclear whether WMH in different anatomical regions play distinct roles in glymphatic dysfunction. Kim et al. divided white matter hyperintensities into juxtaventricular (within 3 mm of the ventricular surface), periventricular (in the marginal zone 3-13 mm between ventricles and cortex), deep (between periventricular and subcortical), and juxtacortical (within 4 mm of the cortical-medullary junction) WMH, representing different pathologies and pathogenic mechanisms (Kim, MacFall, & Payne, 2008). Understanding the relationship between regional WMH and the glymphatic system may help us further understand the mechanisms of glymphatic circulation abnormalities and provide new targets for precision intervention in diseases such as AD.

Recently, Taoka et al. proposed a non-invasive in vivo method called diffusion tensor imaging along the perivascular space (DTI-ALPS) for non-invasively assessing human glymphatic function (Taoka et al., 2017). DTI-ALPS evaluates the movement of water molecules along perivascular spaces in different directions by measuring diffusivity along deep medullary veins at the level of the lateral ventricular body, which is a key process in brain glymphatic circulation. Furthermore, a study has found a significant correlation between the ALPS index and cerebrospinal fluid clearance function assessed by contrast imaging, supporting its validity as a surrogate marker of brain glymphatic function (Zhang et al., 2021). Therefore, DTI-ALPS has become a valuable tool for non-invasively assessing glymphatic system function and has been applied to various diseases such as Parkinson’s disease (Shen et al., 2022), frontotemporal dementia (Jiang et al., 2023), AD (Huang et al., 2024), and neuromyelitis optica (Cacciaguerra et al., 2022). Studies have shown that the ALPS index is reduced in AD patients compared with normal controls (Hsu et al., 2023) and is related to cognitive function. Some studies have also found that the ALPS index is associated with amyloid and tau deposition in the brain (Hsu et al., 2023; Ota et al., 2022), potentially predicting amyloid deposition, neurodegeneration, and clinical progression in AD (Huang et al., 2024). However, the relationship between the plasma biomarker Aβ and glymphatic function remains unclear.

This study aims to investigate: (1) Changes in the ALPS index across the AD clinical spectrum from HC to AD, and the correlations between CSVD, plasma biomarkers, cognitive function, and the ALPS index; (2) Whether the ALPS index mediates the relationship between plasma Aβ and cognitive performance. We used WMH burden to represent CSVD. We hypothesized that both WMH and plasma Aβ have independent associations with brain glymphatic function, and that the ALPS index may mediate the relationship between plasma Aβ levels and cognitive function.

## Materials and methods

### Study participants

This study was approved by the Ethics Committee of Hainan General Hospital (Affiliated Hainan Hospital of Hainan Medical University). A total of 258 subjects were initially recruited. Eleven subjects were excluded due to incomplete diffusion tensor imaging (DTI) data, 10 subjects were excluded due to incomplete neuropsychological assessments, 9 subjects were excluded due to obvious brain lesions such as infarction or tumors, and 12 subjects were excluded due to excessive head motion. Finally, a total of 216 subjects were included in this study.

### Neuropsychological assessment

In this study, global cognition was assessed using the Mini-Mental State Examination (MMSE). The MMSE is a 30-item screening tool used to evaluate cognitive abilities, including orientation, memory, attention, and language abilities. The total MMSE score represents the participant’s overall cognitive function, with higher scores indicating better global cognitive performance. Executive function was assessed using the Shape Trail Test A (STT-A), in which participants were asked to connect numbers in ascending order by drawing lines as quickly as possible. The completion time (in seconds) was used for analysis, with a longer time indicating worse executive performance. Memory function was evaluated using the Rey Auditory Verbal Learning Test (RAVLT). Participants were asked to recall a 15-word list over repeated trials and again after a delay. In the present study, long-term delayed free recall (5 minutes, AVLT-N5) and the total number of words recalled after 20 minutes (AVLT-N7) were used as memory indicators, with higher scores indicating better memory performance. Language function was assessed using the semantic verbal fluency test (VFT) and Boston Naming Test (BNT). For the VFT, participants were asked to list as many animal names as possible within 60 seconds; the total number of correct responses was used for analysis, with higher scores indicating better semantic fluency. For the BNT, participants were asked to name 30 objects/items printed in a book, with higher scores indicating better language performance. In the present study, all neuropsychological test scores were analyzed as continuous variables. Because normative values may vary by age, education, and population, fixed normal ranges were not used for the primary statistical analyses.

### MRI data acquisition

All participants underwent magnetic resonance imaging using a 3.0 T magnetic resonance imaging (MRI) scanner (Prisma, Siemens, Germany) with a 64-channel head–neck receiver coil. The imaging protocol included three-dimensional magnetization prepared rapid acquisition gradient echo (MPRAGE) sequences for anatomical imaging. Specific scanning parameters were as follows: (1) MPRAGE: echo time (TE) = 2.26 ms; repetition time (TR) = 2300 ms; inversion time = 900 ms; flip angle = 8°; slice thickness = 1 mm; field of view (FOV) = 256 × 256 mm^2^; voxel size = 1 × 1 × 1 mm^3^. (2) DTI scan parameters: number of directions = 64; TE = 65.0 ms; TR = 4,500 ms; number of slices = 74; FOV = 244 × 244 mm^2^; slice thickness = 2 mm; DTI data were acquired through multi-slice simultaneous acquisition echo-planar imaging sequences, including 64 diffusion gradient directions (*b* = 1,000 s/mm^2^) and 12 *b* = 0 s/mm^2^ images.

### Blood biomarker analysis

We collected 2 ml of venous blood in tubes containing ethylenediaminetetraacetic acid and obtained plasma supernatant after centrifuging samples at 2500 g/min for 15 minutes at 4 °C. All plasma samples were stored at −80 °C and thawed immediately on ice before analysis. Plasma levels of Aβ and GFAP were measured using the single molecule array (Simoa™) platform (Quanterix Corporation, Billerica, MA, USA), with experiments strictly following manufacturer’s instructions. Simoa assays used the following commercial kits: β-amyloid (1–42) and β-amyloid (1–40), Simoa™ β-Amyloid (1–42) and β-Amyloid (1–40) Advantage Kits; GFAP, Simoa™ GFAP Discovery Kit. All assays were performed in duplicate, with mean concentration values reported. For samples with concentrations below the lower limit of quantification, concentrations were set to half the lower limit of quantification.

### ALPS index calculation

The ALPS index is calculated by comparing diffusivity along the perivascular space (PVS) direction at the lateral ventricular body level with the diffusivity of projection and association fibers (Supplementary Figure S1). At this level, the PVS direction is perpendicular to the ventricular wall, and is thus defined as the right–left direction (*x*-axis). This direction is also perpendicular to the directions of projection fibers (*z*-axis) and association fibers (*y*-axis). Therefore, diffusivity along the *x*-axis and the diffusivity of projection and association fibers can partially represent diffusivity along the PVS. To quantify glymphatic system activity, the ALPS index is defined as follows:

(1) $$\text{ALPS index}=\frac{\text{mean}\left(D_{\text{xproj}},D_{\text{xassoc}}\right)}{\text{mean}\left(D_{\text{yproj}},D_{\text{zassoc}}\right)},$$

where $D_{\text{xproj}}$ and $D_{\text{yproj}}\;$ represent *x* − axis and *y* − axis diffusivity in projection fiber regions, and $D_{\text{xassoc}}$ and $D_{\text{zassoc}}$ represent *x*-axis and *z*-axis diffusivity in association fiber regions. A higher ALPS index indicates better glymphatic system function.

Diffusion metric images, including $D_{x}$ , $D_{y}$ , $D_{z}$ , and fractional anisotropy (FA) maps, were generated using DSI Studio software (https://dsi-studio.labsolver.org/). To avoid bias from manual region of interest (ROI) drawing, this study employed a template-based algorithm (Yokota et al., 2019), which minimizes operator-dependent variability and enhances reproducibility. Briefly, each subject’s FA map was nonlinearly registered to the ICBM-DTI-81 template FA map using SPM12 software (Wellcome Trust Centre of Cognitive Neurology, University College London, UK, http://www.fil.ion.ucl.ac.uk/spm/). Registration accuracy was further confirmed through manual inspection. Other diffusion metric images from individual subjects were transformed according to the FA map registration matrix. Additionally, left and right brain projection fiber and association fiber ROIs were extracted based on template labels. ROI range in the cranial-caudal direction was limited to the area where the x-axis line is perpendicular to the lateral ventricular body. According to formula (1), ALPS indices for the left and right hemispheres were automatically calculated.

### Statistical analysis

All statistical analyses were performed using SPSS 19.0 (SPSS Inc., Chicago) and R software version 3.5.2 (R Core Team, www.R-project.org). Continuous and categorical variables were expressed as mean ± standard deviation and number/frequency (%), respectively. Analysis of variance (ANOVA) and chi-square tests were used to compare demographic characteristics between groups, with post-hoc Bonferroni multiple comparisons to examine between-group differences in HC, SCD, MCI, and AD. Using age, sex, education level, and APOE 4 carrier status as covariates, partial correlation analysis was employed to analyze independent relationships between plasma Aβ levels, regional WMH, and the ALPS. We used the PROCESS plugin in SPSS 19.0 (SPSS Inc., Chicago) for mediation analysis. Statistical significance was set at *P* < 0.05 for all analyses. To account for multiple comparisons, the Benjamini–Hochberg false discovery rate (FDR) procedure was applied to all correlation analyses. For plasma amyloid measures, FDR correction was applied only within that set of variables. *P* values reported in the results and figures reflect FDR-corrected values unless otherwise noted.

## Results

Table 1 shows the characteristics of participants in each group. The four groups showed no significant differences in age, sex, and APOE4 carrier status (all *P* > 0.05). Education levels in the MCI and AD groups were lower than those in the HC and SCD groups. MMSE scores in the MCI and AD groups were significantly lower than in the HC (*P* = 0.003; *P* < 0.001) and SCD (*P* < 0.001; *P* < 0.001) groups, and the AD group had lower MMSE scores than the MCI group (*P* < 0.001). Plasma Aβ42/Aβ40 ratios in AD patients were significantly lower than those in the HC and SCD groups (*P* = 0.001; *P* < 0.001), and the MCI group plasma Aβ42/Aβ40 ratios were significantly lower than in the SCD group (*P* = 0.017). There were significant differences in Aβ42 levels among the four groups, with the AD group significantly lower than the SCD group (*P* = 0.006). Plasma GFAP in the AD group was significantly higher than in the HC, SCD, and MCI groups (*P* = 0.006; *P* = 0.003; *P* = 0.024). Compared with the HC and SCD groups, the MCI group showed significantly reduced N5 and N7 scores (all *P* < 0.001); compared with the HC, SCD, and MCI groups, the AD group showed significantly reduced N5 and N7 scores (all *P* < 0.001). Compared with the SCD group, the MCI group performed worse on STT-A (*P* = 0.001). The AD group had a longer time in completing the STT-A than the HC, SCD, and MCI groups (all *P* < 0.001). Compared with the HC and SCD groups, the MCI group showed significantly reduced VFT and BNT scores (all *P* < 0.001); compared with the HC, SCD, and MCI groups, the AD group showed significantly reduced VFT and BNT scores (all *P* < 0.001). These overall and cognitive domain differences remained significant after adjusting for education level. Compared with the HC, SCD, and MCI groups, the AD group showed significantly higher log-transformed juxtaventricular WMH (lgjuxWMH) burdens (all *P* < 0.05). The MCI group also had higher juxWMH levels than the SCD group (*P* < 0.05). Compared with the HC and SCD groups, the AD group showed significantly higher log-transformed periventricular WMH (lgpWMH) burdens (all *P* < 0.05). The four groups did not show a statistically significant difference regarding log-transformed deep WMH (lgdWMH) and log-transformed juxtacortical WMH (lgjcWMH) (all *P* > 0.05).

**Table 1.** Demographic and clinical characteristics of participants Table 1. long description.

	HC	SCD	MCI	AD	F/χ^2^	P值
Number	33	51	93	39	/	/
Age (years)	66.61 ± 8.34	67.78 ± 5.77	67.65 ± 5.79	70.31 ± 8.74	4.04	0.257
Education (years)	13.82 ± 4.24	15.20 ± 3.24	10.96 ± 3.79^**a**^	10.77 ± 4.08^**bd**^	46.79	**<0.001**
Sex (F/M)	17/16	36/15	54/39	22/17	3.71	0.294
APOE4, *n* (%)	4 (12.5)	9 (18.0)	26(28.3)	14(36.8)	7.38	0.062
Aβ42	5.58 ± 1.38	5.91 ± 1.59	5.42 ± 1.40	4.87 ± 1.41^**d**^	3.81	**0.011**
Aβ40	84.38 ± 16.78	87.55 ± 12.47	90.50 ± 17.82	89.30 ± 19.06	1.08	0.360
Aβ42/ Aβ40	0.07 ± 0.01	0.07 ± 0.01	0.06 ± 0.01^**c**^	0.05 ± 0.01^**bd**^	8.69	**<0.001**
GFAP	91.81 ± 40.66	96.77 ± 54.76	111.93 ± 81.66	152.45 ± 87.15^**bde**^	5.42	**0.001**
MMSE	28.41 ± 1.86	28.42 ± 1.76	25.71 ± 2.81^**ac**^	18.71 ± 6.58^**bde**^	64.29	**<0.001**
N5	7.17 ± 2.48	6.76 ± 2.49	3.54 ± 2.44^**ac**^	2.03 ± 2.57^**bde**^	41.80	**<0.001**
N7	22.28 ± 1.89	21.84 ± 2.29	19.40 ± 3.12^**ac**^	15.22 ± 5.11^**bde**^	36.52	**<0.001**
VFT	21.59 ± 5.56	20.54 ± 5.10	14.88 ± 5.15^**ac**^	9.77 ± 4.30^**bde**^	46.50	**<0.001**
BNT	26.41 ± 2.75	27.32 ± 2.32	22.90 ± 5.00^**ac**^	18.08 ± 5.34^**bde**^	38.36	**<0.001**
STT-A	67.89 ± 24.55	63.60 ± 15.91	93.46 ± 44.29^**c**^	167.80 ± 77.77^**bde**^	41.73	**<0.001**
lgjuxWMH	0.37 ± 0.21	0.33 ± 0.22	0.45 ± 0.25^**c**^	0.60 ± 0.25^**bde**^	10.75	**<0.001**
lgpWMH	−0.42 ± 0.60	−0.50 ± 0.66	−0.27 ± 0.74	0.09 ± 0.87^**bd**^	5.18	**0.002**
lgdWMH	−0.30 ± 0.37	−0.30 ± 0.50	−0.23 ± 0.57	−0.051 ± 0.78	1.63	0.184
lgjcWMH	−0.60 ± 0.34	−0.57 ± 0.45	−0.45 ± 0.43	−0.32 ± 0.73	2.58	0.055

*Note:* Values are expressed as mean ± standard deviation. Bold *P* values indicate significant intergroup differences (ANOVA, *P* < 0.05); post-hoc two-sample *t*-tests were subsequently performed with Bonferroni correction (*P* < 0.05). HC, healthy controls; SCD, subjective cognitive decline; MCI, mild cognitive impairment; AD, Alzheimer’s disease; MMSE, Mini-Mental State Examination; GFAP, glial fibrillary acidic protein; juxWMH, juxtaventricular white matter hyperintensities; pWMH, periventricular white matter hyperintensities; dWMH, deep white matter hyperintensities; jcWMH, juxtacortical white matter hyperintensities.

**^a^** Indicates intergroup differences between HC and MCI groups.

^**b**^ Indicates intergroup differences between HC and AD groups.

^**c**^ Indicates intergroup differences between SCD and MCI groups.

**^d^** Indicates intergroup differences between SCD and AD groups.

^**e**^ Indicates intergroup differences between MCI and AD groups.

### Between-group ALPS differences

**Figure 1.**
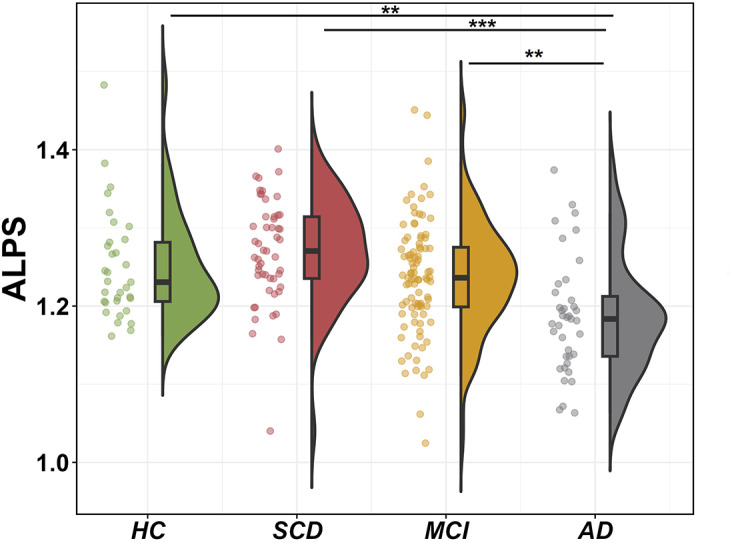
Between-group ALPS differences. This figure shows between-group differences in ALPS. The AD group had significantly lower ALPS compared to HC, SCD, and MCI groups (all *P* < 0.01); the MCI group showed a trend toward lower ALPS than the SCD group (*P* = 0.058, Bonferroni-corrected; *P* = 0.01 uncorrected). There were no statistical differences between other groups. *Note:* ALPS, ‘analysis of diffusion tensor imaging along the perivascular space’; HC, ‘healthy controls’; SCD, ‘subjective cognitive decline’; MCI, ‘mild cognitive impairment’; AD, ‘Alzheimer’s disease’. Figure 1. long description.

There were statistically significant differences in the ALPS index between the four groups (*P* < 0.001), showing a gradual declining trend from HC, through SCD and MCI, to AD (Figure 1). The AD group had significantly lower ALPS index values compared with the HC, SCD, and MCI groups (all *P* < 0.001); compared with the SCD group, the MCI group tended to have a lower ALPS index (*P* = 0.058, Bonferroni-corrected; *P* = 0.01 uncorrected). There were no statistical differences between other groups.

### Relationship between ALPS and cognitive function

**Figure 2.**
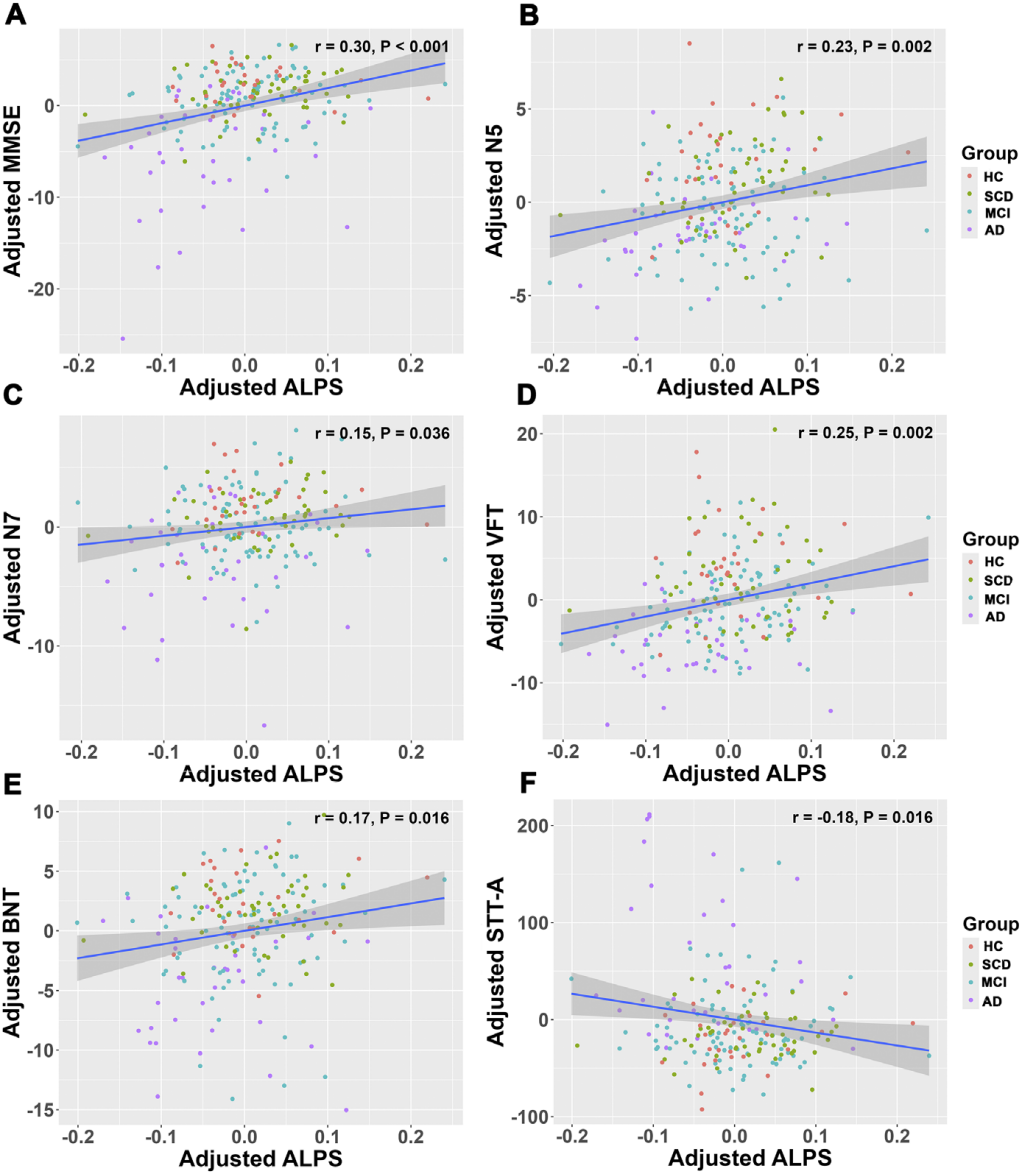
Association between ALPS and cognitive function (regressed for gender, age, education level, and APOE4). After adjusting for gender, age, education level, and APOE4 carrier status, ALPS was positively correlated with MMSE (*r* = 0.30, *P* < 0.001), as well as with N5 and N7 (*r* = 0.23, *P* = 0.002; *r* = 0.15, *P* = 0.036). It was also positively correlated with VFT and BNT scores (*r* = 0.25, *P* = 0.002; *r* = 0.17, *P* = 0.016), and negatively correlated with STT-A scores (*r* = −0.18, *P* = 0.016). *Note:* ALPS, ‘Analysis of diffusion tensor imaging along the perivascular space’; HC, ‘Healthy Controls’; SCD, ‘Subjective Cognitive Decline’; MCI, ‘Mild Cognitive Impairment’; AD, ‘Alzheimer’s Disease’; MMSE, ‘Mini-Mental State Examination’; VFT, ‘verbal fluency test’; BNT, ‘Boston naming test’; STT-A, ‘Shape Trail Test A’. Figure 2. long description.

After adjusting for sex, age, education level, and APOE4 carrier status, the ALPS index was positively correlated with MMSE (*r* = 0.30, *P* < 0.001), as well as with N5 and N7 (*r* = 0.23, *P* = 0.002; *r* = 0.15, *P* = 0.036). It was also positively correlated with VFT and BNT scores (*r* = 0.25, *P* = 0.002; *r* = 0.17, *P* = 0.016), and negatively correlated with STT-A scores (*r* = −0.18, *P* = 0.016). (Figure 2).

### Relationship between ALPS and plasma biomarkers

**Figure 3.**
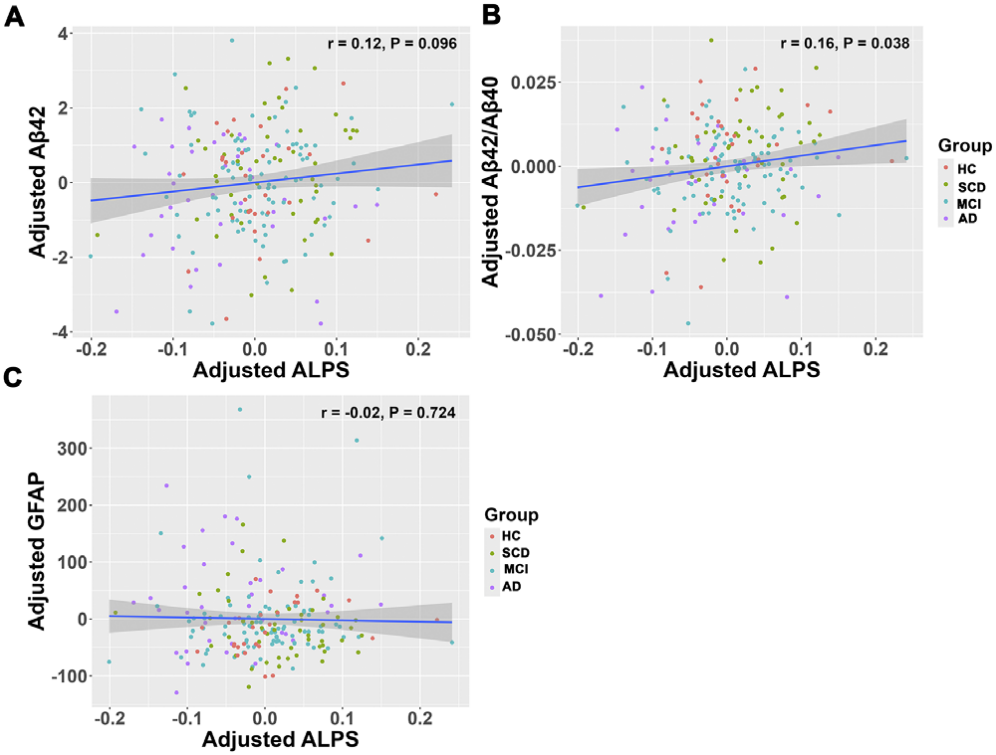
Association between ALPS and plasma biomarkers. After regressing for gender, age, education level, and APOE4 carrier status, there was no significant correlation between ALPS and Aβ42 (*r* = 0.12, *P* = 0.096); ALPS was positively correlated with Aβ42/Aβ40 levels (*r* = 0.16, *P* = 0.038). There was no significant correlation between ALPS and GFAP (*r* = −0.02, *P* = 0.724). *Note:* ALPS, ‘Analysis of diffusion tensor imaging along the perivascular space’; HC, ‘Healthy Controls’; SCD, ‘Subjective Cognitive Decline’; MCI, ‘Mild Cognitive Impairment’; AD, ‘Alzheimer’s Disease’; MMSE, ‘Mini-Mental State Examination’. Figure 3. long description.

After adjusting for sex, age, education level, and APOE4 carrier status, the correlation between the ALPS index and Aβ42 was not significant (*r* = 0.12, *P* = 0.096), but the ALPS index was positively correlated with Aβ42/Aβ40 ratios (*r* = 0.16, *P* = 0.038). The ALPS index had no significant correlation with GFAP (*r* = −0.02, *P* = 0.724) (Figure 3).

### Relationship between ALPS and regional white matter hyperintensities

**Figure 4.**
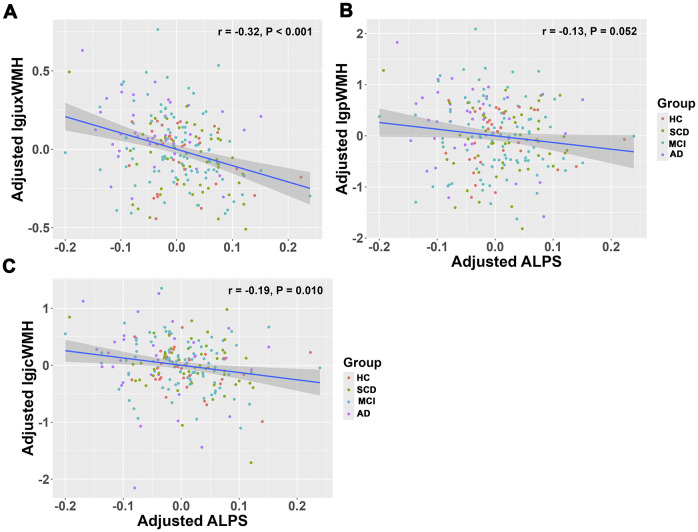
Association between ALPS and white matter hyperintensities in different regions. After adjusting for gender, age, education level, and APOE4 as covariates, ALPS showed significant negative correlations with lgjuxWMH and lgjcWMH (*r* = −0.32, *P* < 0.001; *r* = −0.19, *P* = 0.010); the correlation between ALPS and lgpWMH approached statistical significance (*r* = −0.13, *P* = 0.052). *Note:* ALPS, ‘Analysis of diffusion tensor imaging along the perivascular space’; HC, ‘Healthy Controls’; SCD, ‘Subjective Cognitive Decline’; MCI, ‘Mild Cognitive Impairment’; AD, ‘Alzheimer’s Disease’; juxWMH, ‘juxtaventricular white matter hyperintensities’; pWMH, ‘periventricular white matter hyperintensities’; jcWMH, ‘juxtacortical white matter hyperintensities’. Figure 4. long description.

After adjusting for sex, age, education level, and APOE4 carrier status as covariates, the ALPS index was significantly negatively correlated with lgjuxWMH and lgjcWMH (*r* = −0.32, *P* < 0.001; *r* = −0.19, *P* = 0.010); the correlation between the ALPS index and lgpWMH approached significance (*r* = −0.13, *P* = 0.052) (Figure 4).

### ALPS index as a mediator between plasma Aβ42/Aβ40 ratio and cognitive performance

**Figure 5.**
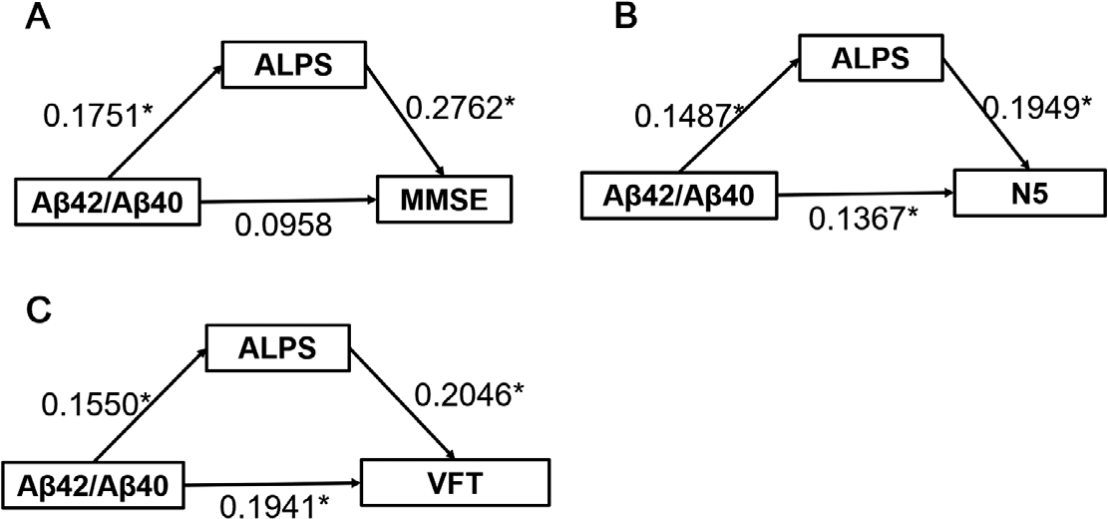
Mediating effect of ALPS in the relationship between plasma Aβ42/Aβ40 and cognitive performance. *Note:* ALPS, ‘Analysis of diffusion tensor imaging along the perivascular space’; MMSE, ‘Mini-Mental State Examination’; VFT, ‘verbal fluency test’. Figure 5. long description.

Using sex, age, education level, and APOE4 carrier status as covariates, mediation effect analysis revealed that the ALPS index showed significant mediation effects on the relationships between plasma Aβ42/Aβ40 ratio and overall cognitive function (indirect effect *β* = 0.0484, *P* < 0.05), memory function as representing by N5 (indirect effect *β* = 0.0290, *P* < 0.05), and language function (indirect effect *β* = 0.0317, *P* < 0.05) (Figure 5).

## Discussion

This study examined ALPS indices across different cognitive status groups and found that AD patients exhibited significantly lower ALPS compared with HC, SCD, and MCI groups, with the MCI group tended to show lower ALPS relative to the SCD group. Further analysis showed that specific regional WMH and the plasma Aβ42/Aβ40 ratio were independently correlated with the ALPS index. Moreover, the ALPS index was significantly correlated with multiple cognitive domains (including overall cognition, memory, executive function, and language ability) and served as a mediator between plasma Aβ levels and cognitive performance. These findings provide important insights into the relationship between glymphatic dysfunction and AD pathophysiology.

Consistent with previous reports (Hsu et al., 2023; Kamagata et al., 2022), our results further confirm that AD patients exhibit impaired glymphatic system function, specifically manifested as a significantly reduced ALPS index. This reduction may reflect reduced brain metabolic waste clearance, which could contribute to AD pathologenesis. Compared with prior studies, the innovation of this study lies in including the SCD group as a control, thereby encompassing the full cognitive spectrum from HC to AD. Through this design, MCI patients tended to have lower ALPS index than the SCD group, although this difference did not reach statistical significance after correction for multiple comparisons, highlighting potential early involvement of glymphatic dysfunction. These results support the potential utility of the ALPS index as a promising candidate biomarker for early AD diagnosis. Previous research by Huang et al. also reported that ALPS decline can be detected in the preclinical stage of AD (Huang et al., 2024), with evidence suggesting that glymphatic dysfunction may precede amyloid pathology (Huang et al., 2024). This temporal relationship provides important insights into AD pathogenesis and opportunities for early intervention.

Our analysis of the correlation between the ALPS index and cognitive function is consistent with multiple previous studies (Kamagata et al., 2022; Steward et al., 2021; Taoka et al., 2017). This broad pattern of correlations suggests that glymphatic system functional impairment may influence neuronal health and contribute to global cognitive decline. Notably, this correlation is not limited to any specific cognitive domain but encompasses multiple domains, indicating that glymphatic system dysfunction may exert widespread effects across diverse brain regions. These findings collectively support glymphatic system dysfunction as a potential contributor to AD progression and provide an empirical basis for using the ALPS index as a biomarker for assessing AD severity and prognosis.

Further analysis showed that the ALPS index was significantly correlated with WMH volumes in specific brain regions, particularly the juxtaventricular, periventricular, and juxtacortical regions. These regions are adjacent to cerebrospinal fluid flow pathways, where the glymphatic system is active, suggesting that white matter structural damage may affect glymphatic function. Juxtaventricular WMH has been associated with cerebrospinal fluid leakage, periventricular WMH is associated with hypoperfusion and ischemic states, while juxtacortical WMH may be closely related to small vessel disease and ischemia-hypoxia (Kim, MacFall, & Payne, 2008). These regions are located in key areas of the brain, typically at the intersection of cerebrospinal fluid and plasma flow. Juxtaventricular and periventricular regions are located around the ventricles and are often pathways for cerebrospinal fluid and metabolic waste elimination through the glymphatic system. Juxtacortical regions are located at the junction between cortex and white matter, and WMH in this location may be related to cerebrospinal fluid flow obstruction in these layers. Therefore, the regional specificity of WMH associations with ALPS suggests that lesions from different mechanisms may all adversely affect glymphatic function.

Notably, despite significant correlations with juxtaventricular, periventricular, and juxtacortical WMH, the ALPS index was not associated with deep WMH. This finding carries important theoretical significance. Deep white matter is located between the periventricular white matter and juxtacortical white matter, and is relatively distant from primary cerebrospinal fluid flow pathways. Deep WMH likely reflects microvascular lesions, small artery sclerosis, or other vascular pathological changes, which may predominantly affect tissue metabolism through local interstitial fluid stasis rather than directly impairing global glymphatic system function. In contrast, WMH in juxtaventricular, periventricular, and juxtacortical regions may be more closely related to cerebrospinal fluid flow dynamics and glymphatic status. This regional difference suggests that WMH in different regions may interact with glymphatic function through different pathological mechanisms: juxtaventricular, periventricular, and juxtacortical WMH may disrupt cerebrospinal fluid flow and waste clearance pathways, while deep WMH primarily reflect vascular-origin lesions with relatively limited direct impact on the glymphatic system. This finding underscores the importance of considering the regional distribution of WMH, rather than total WMH burden alone, when evaluating and addressing glymphatic dysfunction in AD.

This study found that the plasma Aβ42/Aβ40 ratio was significantly correlated with the ALPS index. In recent years, plasma Aβ42/Aβ40 has been recognized as a peripheral biomarker reflecting AD pathology (Janelidze et al., 2021; Schindler et al., 2019). Although Aβ primarily accumulates as β-amyloid plaques in the brain, plasma Aβ levels can also reflect cerebral metabolic changes. Studies show that the plasma Aβ42/Aβ40 relates to brain Aβ deposition, especially in early AD (Janelidze et al., 2016; Pérez-Grijalba et al., 2019) and discriminates abnormal from normal amyloid-β positron emission tomography (Aβ-PET) states (Janelidze et al., 2024). The observed correlation between the plasma Aβ42/Aβ40 ratio and the ALPS index may reflect multi-level pathophysiological associations. On one hand, glymphatic system dysfunction may be associated with reduced Aβ clearance efficiency, potentially increasing brain and plasma Aβ. On the other hand, peripheral Aβ levels may reflect systemic metabolic alterations, which could indirectly affect cerebral blood flow dynamics and glymphatic system function. Taken together, these processes may form a bidirectional feedback loop between glymphatic dysfunction and Aβ pathology, which could potentially contribute to disease progression. However, this remains a hypothesized mechanism and was not directly tested in the present study. Crucially, mediation analysis showed that ALPS significantly mediated the relationships between plasma Aβ42/Aβ40 ratio and overall cognition, memory, and language performance, supporting the functional relevance of glymphatic activity in AD-related cognitive function. These findings provide further evidence that glymphatic dysfunction may contribute to AD pathogenesis and underscore the clinical potential of the ALPS index as a potential biomarker for brain metabolic waste clearance capacity. Future research can further explore the combined application of the ALPS index with other AD-related biomarkers to improve early diagnostic accuracy and longitudinal disease monitoring.

This study has some limitations. First, this study did not include cerebrospinal fluid examination or PET imaging data, limiting our ability to directly assess the relationship between the ALPS index and cerebrospinal fluid biomarkers or Aβ/Tau deposition. This limits a comprehensive understanding of glymphatic dysfunction in relation to classic AD pathology. Second, although the ALPS index has been preliminarily validated through glymphatic MRI studies, its effectiveness in assessing human glymphatic system function still lacks direct pathophysiological evidence. In addition, formal reliability analyses (e.g. intra- or inter-rater reproducibility or test–retest reliability) were not performed, which may affect measurements robustness. Third, the ALPS index, as a global indicator of lymphatic activity, cannot capture regional heterogeneity, and dysfunction in different brain regions may have differential impacts on cognitive function. Fourth, as a cross-sectional study, causal relationships and temporal sequences between ALPS decline, WMH development, and cognitive deterioration cannot be determined, and the sequence of cerebrovascular lesions relative to glymphatic dysfunction should be interpreted cautiously. Fifth, ALPS measurements reflect local diffusion characteristics rather than direct flow. Sixth, this study’s moderate sample size and single-center recruitment may have limited statistical power and generalizability. Future studies should include larger cohorts, multi-center cohorts, and conduct longitudinal studies to track ALPS changes over time. In addition, integrating multimodal imaging with data-driven analytic strategies, including machine learning or deep learning, may help improve the robustness, automation, and biological interpretability of glymphatic-related imaging markers (Cai et al., 2025; Lin et al., 2026; Zhang, Wang, Qi, & Peng, 2024). Finally, key modulators of glymphatic function, including sleep quality, blood pressure variability, and other vascular factors, were not assessed. These unmeasured variables may have influenced the observed associations and should be considered in future studies to more comprehensively evaluate glymphatic function.

## Conclusion

In summary, this study demonstrated that AD patients exhibited significantly lower ALPS indices compared with HC, SCD, and MCI groups, with the MCI group tending to show lower ALPS than the SCD group, indicating that glymphatic dysfunction may already be present even at early stages. Specific regional WMH were negatively correlated with the ALPS index, whereas deep WMH showed no significant association, suggesting regional specificity in the impact of white matter lesions on glymphatic function. The positive association between ALPS and the plasma Aβ42/Aβ40 further underscores the potential role of the glymphatic system in Aβ clearance. Moreover, the widespread correlations of ALPS with multiple cognitive domains and its mediating effect between Aβ and cognitive performance highlight the functional relevance of glymphatic dysfunction in AD pathogenesis. These findings support the use of the ALPS index as a potential biomarker for AD severity and prognosis, and provide a foundation for developing therapeutic strategies targeting the glymphatic system.

## Supporting information

10.1017/S0033291726105005.sm001 Chen et al. supplementary material Chen et al. supplementary material

## Data Availability

The data that support the findings of this study are available on request from the corresponding author when the appropriate data sharing agreements are consented.

## Supplementary material

The supplementary material for this article can be found at http://doi.org/10.1017/S0033291726105005.

## Long descriptions

### Table 1. Long description

The table contains 7 columns: Variable, H C (n=33), S C D (n=51), M C I (n=93), A D (n=39), F or chi-squared, and P value.

Key findings include:

* Age (years): No significant difference across groups (P = 0.257).

* Education (years): Significant differences (P < 0.001), with lower education in M C I (10.96) and A D (10.77) compared to H C (13.82) and S C D (15.20).

* APOE4 presence: Increases from H C (12.5%) to A D (36.8%) (P = 0.062).

* Biomarkers: A beta 42/A beta 40 ratio significantly decreases from H C (0.07) to A D (0.05) (P < 0.001). G F A P levels significantly increase from H C (91.81) to A D (152.45) (P = 0.001).

* Cognitive Scores: M M S E, N 5, N 7, V F T, and B N T all show significant declines (P < 0.001) as cognitive impairment progresses from H C to A D. For example, M M S E drops from 28.41 in H C to 18.71 in A D.

* Imaging: S T T dash A time significantly increases in A D (167.80) compared to H C (67.89). White matter hyperintensities (lgjuxWMH and lgpWMH) show significant increases in the A D group (P < 0.001 and P = 0.002 respectively).

Navigate back to Table 1..

### Figure 1. Long description

The vertical Y-axis is labeled A L P S with numerical increments from 1.0 to 1.4. The horizontal X-axis lists four diagnostic groups from left to right.

* H C (Healthy Controls): Green violin plot with a median A L P S value centered around 1.25.

* S C D (Subjective Cognitive Decline): Red violin plot with a median slightly higher than H C, approximately 1.28.

* M C I (Mild Cognitive Impairment): Yellow violin plot with a median around 1.24.

* A D (Alzheimer’s Disease): Grey violin plot showing the lowest distribution with a median below 1.2.

Each group includes a central box plot and a cluster of individual data points to the left of each violin. Horizontal significance bars at the top of the plot indicate statistical differences. A bar with two asterisks connects H C to A D. A bar with three asterisks connects S C D to A D. A bar with two asterisks connects M C I to A D. These bars indicate that the A D group has significantly lower A L P S scores compared to all other groups.

Navigate back to Figure 1..

### Figure 2. Long description

A multi-panel figure containing six scatter plots labeled A through F. All plots share a common x-axis titled Adjusted A L P S ranging from negative 0.2 to 0.2. Each plot features a blue linear regression line with a gray shaded confidence interval and data points color-coded by group: H C (red), S C D (green), M C I (teal), and A D (purple).

* Panel A: Y-axis is Adjusted M M S E. Shows a positive correlation with r equals 0.30 and P less than 0.001.

* Panel B: Y-axis is Adjusted N 5. Shows a positive correlation with r equals 0.23 and P equals 0.002.

* Panel C: Y-axis is Adjusted N 7. Shows a positive correlation with r equals 0.15 and P equals 0.036.

* Panel D: Y-axis is Adjusted V F T. Shows a positive correlation with r equals 0.25 and P equals 0.002.

* Panel E: Y-axis is Adjusted B N T. Shows a positive correlation with r equals 0.17 and P equals 0.016.

* Panel F: Y-axis is Adjusted S T T-A. Shows a negative correlation with r equals negative 0.18 and P equals 0.016.

A legend to the right of the panels identifies the groups: H C (Healthy Controls), S C D (Subjective Cognitive Decline), M C I (Mild Cognitive Impairment), and A D (Alzheimer’s Disease).

Navigate back to Figure 2..

### Figure 3. Long description

A three-panel multi-plot labeled A, B, and C. All panels share a common x-axis titled Adjusted A L P S ranging from negative 0.2 to 0.2. A legend to the right of panels B and C identifies four groups by color: H C (red), S C D (green), M C I (teal), and A D (purple).

* Panel A: The y-axis is Adjusted A beta 42 ranging from negative 4 to 4. Data points are widely scattered with a blue regression line showing a slight upward trend. Statistical annotation at the top right reads r equals 0.12, P equals 0.096.

* Panel B: The y-axis is Adjusted A beta 42 over A beta 40 ranging from negative 0.050 to 0.025. The blue regression line shows a statistically significant positive linear correlation. Statistical annotation at the top right reads r equals 0.16, P equals 0.038.

* Panel C: The y-axis is Adjusted G F A P ranging from negative 100 to 300. The data points are clustered near the zero line with a nearly flat blue regression line. Statistical annotation at the top right reads r equals negative 0.02, P equals 0.724.

In all panels, the regression lines are shaded with a gray area representing the confidence interval.

Navigate back to Figure 3..

### Figure 4. Long description

A multi-panel figure consisting of three scatter plots labeled A, B, and C. All plots share a common x-axis labeled Adjusted A L P S ranging from minus 0.2 to 0.2. Data points are color-coded by group: H C (red), S C D (green), M C I (teal), and A D (purple).

* Panel A: The y-axis is Adjusted l g j u x W M H ranging from minus 0.5 to 0.5. A blue regression line shows a clear downward linear trend with a grey shaded confidence interval. Statistical values in the top right are r equals minus 0.32, P less than 0.001.

* Panel B: The y-axis is Adjusted l g p W M H ranging from minus 2 to 2. The blue regression line shows a slight downward trend. Statistical values in the top right are r equals minus 0.13, P equals 0.052.

* Panel C: The y-axis is Adjusted l g j c W M H ranging from minus 2 to 1. The blue regression line shows a moderate downward trend. Statistical values in the top right are r equals minus 0.19, P equals 0.010.

A legend to the right of panels B and C identifies the four diagnostic groups.

Navigate back to Figure 4..

### Figure 5. Long description

A multi-panel flowchart with three panels labeled A, B, and C. Each panel features a triangular mediation model with three rectangular nodes.

Panel A:

* The left node is A beta 42 forward slash A beta 40.

* The top-center node is ALPS.

* The right node is M M S E.

* An arrow from A beta 42 forward slash A beta 40 to ALPS is labeled 0.1751 asterisk.

* An arrow from ALPS to M M S E is labeled 0.2762 asterisk.

* A direct arrow from A beta 42 forward slash A beta 40 to M M S E is labeled 0.0958.

Panel B:

* The left node is A beta 42 forward slash A beta 40.

* The top-center node is ALPS.

* The right node is N 5.

* An arrow from A beta 42 forward slash A beta 40 to ALPS is labeled 0.1487 asterisk.

* An arrow from ALPS to N 5 is labeled 0.1949 asterisk.

* A direct arrow from A beta 42 forward slash A beta 40 to N 5 is labeled 0.1367 asterisk.

Panel C:

* The left node is A beta 42 forward slash A beta 40.

* The top-center node is ALPS.

* The right node is V F T.

* An arrow from A beta 42 forward slash A beta 40 to ALPS is labeled 0.1550 asterisk.

* An arrow from ALPS to V F T is labeled 0.2046 asterisk.

* A direct arrow from A beta 42 forward slash A beta 40 to V F T is labeled 0.1941 asterisk.

Navigate back to Figure 5..
