# Supplementary material for: Glymphatic dysfunction associates with regional white matter hyperintensities and plasma amyloid-β burden across the Alzheimer’s disease continuum
Source: Psychol Med. 2026 Jul 7;56:e220. doi: 10.1017/S0033291726105005 (PMC13370184; doi:10.1017/S0033291726105005)
Supplement: Chen et al. supplementary material [file S0033291726105005sup001.zip › Supplementary Material.docx]

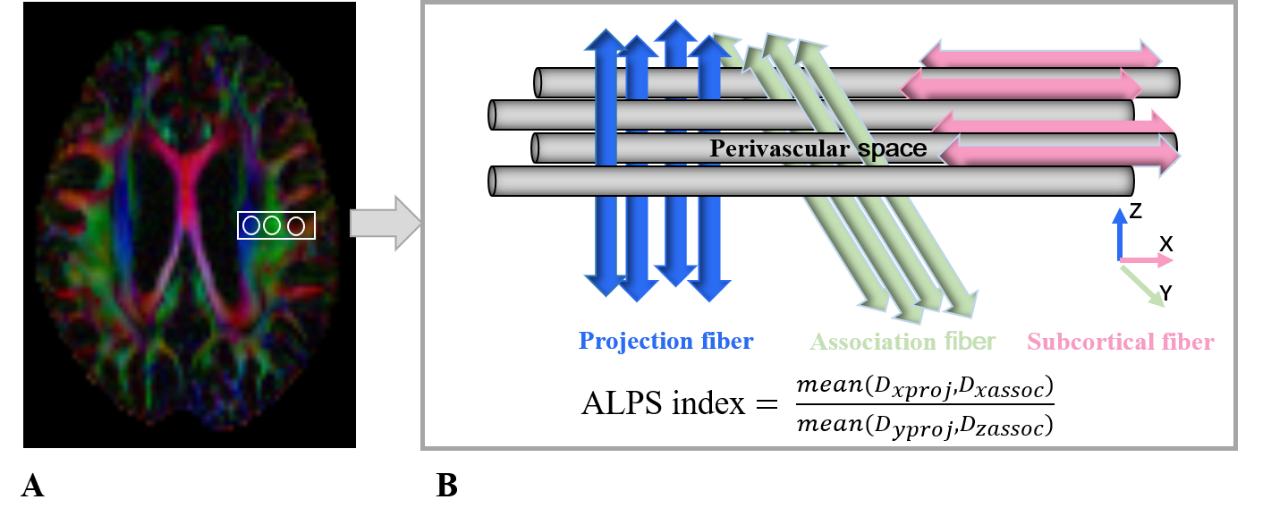


**Supplementary Figure 1 ALPS Index Calculation** (A) Showing the spatial orientation of projection fibers (z-axis: blue), association fibers (y-axis: green), and subcortical fibers (x-axis: red). Three ROIs are positioned within the regions dominated by projection fibers (projection area), association fibers (association area), and subcortical fibers (subcortical area) to assess diffusivities along the three directions (x, y, z). (B) Diagram of the ALPS index, depicting the relationship between the orientation of the perivascular space (gray cylinder) and the fiber directions in the left hemisphere. The perivascular space is oriented perpendicular to both projection and association fibers. The ALPS index was derived by dividing the average of x-axis diffusivity in the projection area ($D_{xproj}$) and the x-axis diffusivity in the association area ($D_{xassoc}$) by the average of y-axis diffusivity in the projection area ($D_{yproj}) a$nd z-axis diffusivity in the association area ($D_{zassoc}$).
