# Supplementary figures and images for: Glymphatic dysfunction associates with regional white matter hyperintensities and plasma amyloid-β burden across the Alzheimer’s disease continuum
Source: Psychol Med. 2026 Jul 7;56:e220. doi: 10.1017/S0033291726105005 (PMC13370184; doi:10.1017/S0033291726105005)

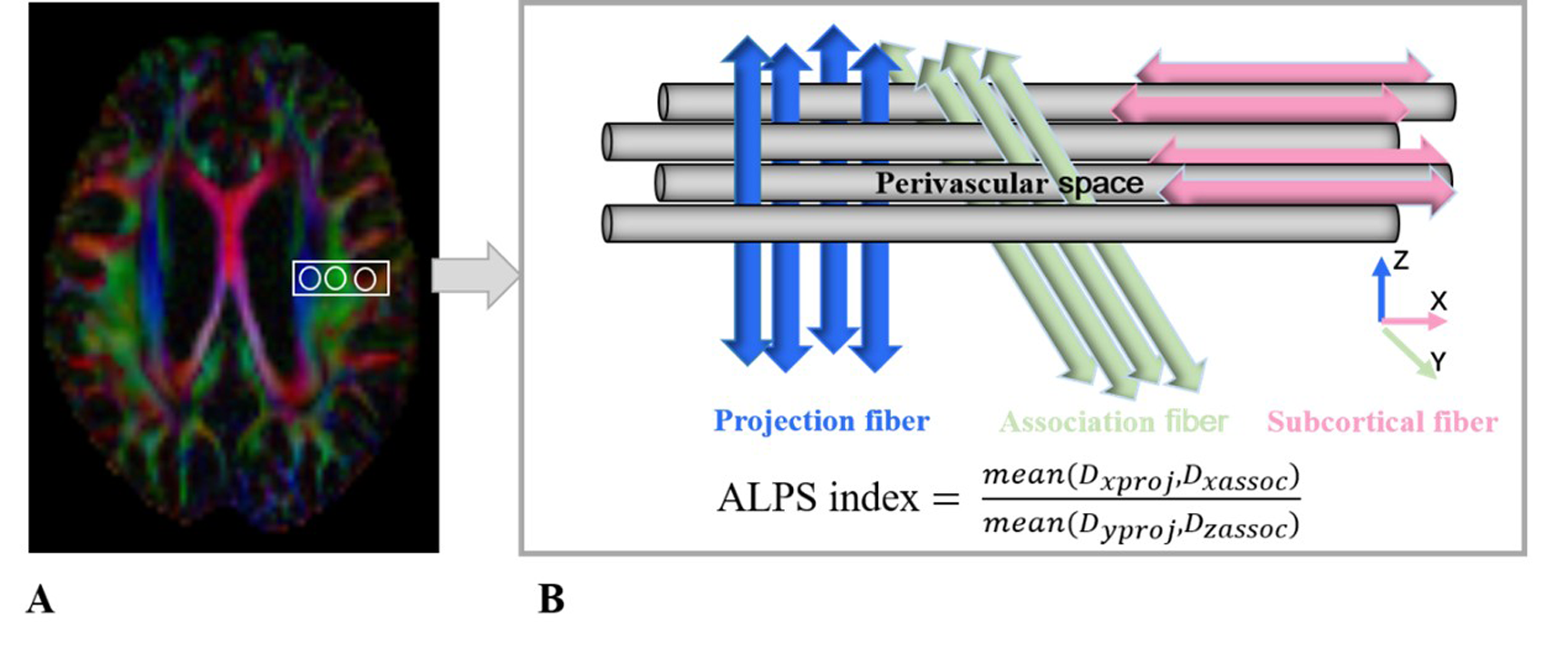

Supplement: Chen et al. supplementary material [file S0033291726105005sup001.zip › Supplementary Figure 1.tif]

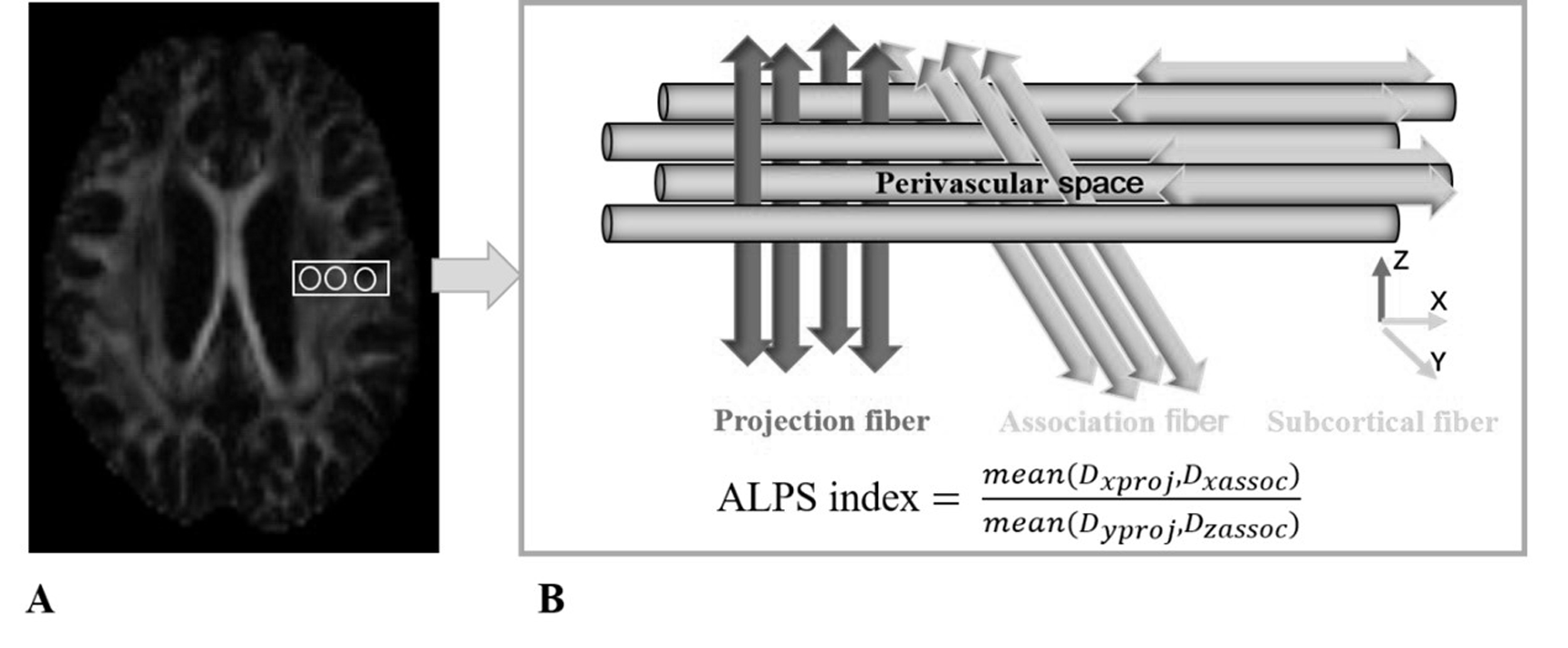

Supplement: Chen et al. supplementary material [file S0033291726105005sup001.zip › Supplementary Figure 1_black-and-white .tif]
